# DSP-PP Precursor Protein Cleavage by Tolloid-Related-1 Protein and by Bone Morphogenetic Protein-1

**DOI:** 10.1371/journal.pone.0041110

**Published:** 2012-07-17

**Authors:** Helena H. Ritchie, Colin T. Yee, Xu-na Tang, Zhihong Dong, Robert S. Fuller

**Affiliations:** 1 Department of Cariology, Restorative Sciences and Endodontics, School of Dentistry, University of Michigan, Ann Arbor, Michigan, United States of America; 2 Department of Endodontology, School of Stomatology, Nanjing University Medical Center, Nanjing, China; 3 Department of Biological Chemistry, University of Michigan Medical School, Ann Arbor, Michigan, United States of America; Russian Academy of Sciences, Institute for Biological Instrumentation, Russian Federation

## Abstract

Dentin sialoprotein (DSP) and phosphophoryn (PP), acidic proteins critical to dentin mineralization, are translated from a single transcript as a DSP-PP precursor that undergoes specific proteolytic processing to generate DSP and PP. The cleavage mechanism continues to be controversial, in part because of the difficulty of obtaining DSP-PP from mammalian cells and dentin matrix. We have infected Sf9 cells with a recombinant baculovirus to produce large amounts of secreted DSP-PP_240_, a variant form of rat DSP-PP. Mass spectrometric analysis shows that DSP-PP_240_ secreted by Sf9 cells undergoes specific cleavage at the site predicted from the N-terminal sequence of PP extracted from dentin matrix: SMQG^447^↓D^448^DPN. DSP-PP_240_ is cleaved after secretion by a zinc-dependent activity secreted by Sf9 cells, generating DSP_430_ and PP_240_ products that are stable in the medium. DSP-PP processing activity is constitutively secreted by Sf9 cells, but secretion is diminished 3 days after infection. Using primers corresponding to the highly conserved catalytic domain of *Drosophila melanogaster* tolloid (a mammalian BMP1 homolog), we isolated a partial cDNA for a *Spodopotera frugiperda* tolloid-related-1 protein (TLR1) that is 78% identical to *Drosophila* TLR1 but only 65% identical to *Drosophila* tolloid. *Tlr1* mRNA decreased rapidly in Sf9 cells after baculovirus infection and was undetectable 4d after infection, paralleling the observed decrease in secretion of the DSP-PP_240_ processing activity after infection. Human BMP1 is more similar to Sf9 and *Drosophila* TLR1 than to tolloid, and Sf9 TLR1 is more similar to BMP1 than to other mammalian homologs. Recombinant human BMP1 correctly processed baculovirus-expressed DSP-PP_240_ in a dose-dependent manner. Together, these data suggest that the physiologically accurate cleavage of mammalian DSP-PP_240_ in the Sf9 cell system represents the action of a conserved processing enzyme and support the proposed role of BMP1 in processing DSP-PP in dentin matrix.

## Introduction

Dentin sialoprotein (DSP) and phosphophoryn (PP) are two major noncollagenous dentin proteins derived from a single copy of DSP-PP gene (also referred to as DSPP) whose expression is tightly regulated during dentinogenesis [1]. DSP-PP transcripts are also expressed in other tissues, including inner ear and jaw tissue [2], [3]. The recent demonstration of DSP-PP promoter-driven lacZ expression in multiple tissues, such as bone, kidney [4], hair follicles [5], [6], salivary gland, and lung (Ritchie unpublished data), further suggests that DSP and PP may have physiological roles in several other organs besides teeth.

It is believed that DSP-PP precursor proteins are the immediate translation products of DSP-PP mRNA (here we use “DSP-PP” to refer generically to the precursor secreted in dentin matrix and “DSP-PP_240_” to refer specifically to the shorter variant form of rat DSP-PP used in these experiments.). DSP-PP undergoes multiple post-translational modifications, including signal peptide cleavage, Asn-linked glycosylation, phosphorylation and proteolytic processing between the DSP and PP domains, to produce mature DSP and PP proteins required for dentin mineralization. Recent research on DSP-PP cleavage has focused on the identification of its initial cleavage site and the protease(s) responsible for cleavage. For example, Qin et al. reported Y438 as the major cleavage site (2001) using tryptic fragments from native, purified DSP [7]. Qin and colleagues proposed that a transmembrane endopeptidase, Phex, was the protease responsible for processing of both DMP1 (e.g., dentin matrix protein 1) and DSP-PP [8], [9]. More recently, Sun et al. reported that a D448A mutation blocked cleavage of recombinant mouse DSP-PP in a cultured human cell system and concluded that the key cleavage site is G^447^↓D^448^ [10]. However, the exact cleavage site of the wild type (wt) mouse DSP-PP precursor in cultured cells was not determined by direct sequencing or mass spectrometry (MS); rather identification of cleavage products relied on gel mobility of immuno-stained or Stains-All-stained bands separated by SDS-PAGE. Thus, the D448A mutant also did not provide direct evidence that G^447^↓D^448^ was the cleavage site.

Other attempts to localize the initial DSP-PP cleavage site have met with limited success because of the lack of sufficient DSP-PP precursor protein available for quantitative studies. Steiglitz et al. proposed in 2004 bone morphogenetic protein-1 (BMP1) as the candidate enzyme for proteolytic cleavage of both DMP1 and DSP-PP [11]. This work demonstrated that BMP1 was responsible for DMP1 cleavage but did not test DSP-PP cleavage because the DSP-PP precursor protein was not available [11]. Recently, Fisher and co-workers identified a mouse DSP-PP precursor protein by expression in transfected human colon carcinoma LoVo cells which lack furin and therefore should be defective in BMP1 activation [12]. LoVo cells were shown to secrete an intact mouse DSP-PP precursor that could be cleaved by exogenously added BMP1 and by the homologous proteins tolloid-like 1 (TLL1) and tolloid-like 2 (TLL2) to generate a DSP-sized band, as shown by Western blot analysis [12]. However, because of the low amounts of DSP-PP and DSP that were detected, no mass spectrometry or N-terminal sequence data were available to identify the precise cleavage site. A mutant form of mouse DSP-PP (with a substitution equivalent to M^445^Q^446^ to I^445^E^446^ in rat DSP-PP_240_) was not cleaved by BMP1, TLL1 or TLL2. Again, analysis of this mutant did not provide direct evidence that G^447^↓D^448^ was the cleavage site. Yamakoshi and co-workers [13], showed that a DSP-PP preparation obtained from porcine dentin matrix was cleaved by the matrix metalloproteinases MMP-2 and MMP-20 at multiple sites making them unlikely candidates for processing enzymes. More recently, similar preparations were shown to be cleaved by BMP1 and MEP1A to generate a product similar in mobility to PP [14]. Taken together, despite intensive investigations since 2001, there is no direct evidence proving that G^447^↓D^448^ is the DSP-PP cleavage site and only Western blot analysis to substantiate the claim that BMP1 can correctly cleave DSP-PP.

Previously, we used a baculovirus expression system that was capable of producing high yields of DSP-PP precursor protein. The secreted DSP-PP_240_ protein could be visualized with Stains-All staining and could be identified unambiguously by mass spectrometry [15]. From MS/MS analysis of isolated tryptic fragments of the PP_240_ band, we proposed that the DSP-PP cleavage site was G^447^↓D^448^ [15]. In this report, we used MS and MS/MS to analyze a smaller, chymotryptic fragment of the PP_240_ band which has permitted direct determination of the amino acid sequence of this peptide by ion trap/fragmentation MS and firmly establishes that the initial cleavage site in DSP-PP_240_ is G^447^↓D^448^.

We also show that cleavage of DSP-PP_240_ at this site occurs after secretion into the conditioned medium of Sf9 cells and that cleavage is catalyzed by an endogenous Zn-dependent proteolytic activity secreted by Sf9 cells. Secretion of this activity is suppressed by baculoviral infection. We show, furthermore, that Sf9 cells transcribe a tolloid-related-1 (TLR1) peptidase gene (*Spodoptera frugiperda tlr1*) and that expression of this gene is similarly suppressed by baculoviral infection. We show that the human homolog of tolloid-related-1, BMP1, can also cleave DSP-PP_240_ to release DSP_430_ and PP_240_.

## Materials and Methods

### Mass Spectrometric (MS) Analysis of PP_240_ to Determine the Cleavage Site in Secreted DSP-PP_240_

PP_240_ was partially purified by the polyanion extraction procedure as previously described [15]. Briefly, conditioned medium (5 ml) was collected from 1×10^6^ Sf9 cells (Novagen, Madison, WI) 4 days after infection with baculovirus containing the DSP-PP_240_ cDNA (hereafter termed DSP-PP_240_ virus) [15]. Trichloroacetic acid (TCA) was added to the medium to a final concentration of 5% (w/v) and incubated at room temperature for 2 min to precipitate the majority of culture medium proteins. The TCA-soluble fraction, collected after centrifugation in an Eppendorf rotor at 12,000 rpm at room temperature for 5 min, was neutralized with one-fifth volume of 3 M Tris-HCl, pH 8.8, and precipitated with one-tenth volume of 1 M CaCl_2_. The calcium precipitate, collected after centrifugation in an Eppendorf rotor at 12,000 rpm for 5 min at room temperature, was dissolved in 1 ml of 5% trichloroacetic acid, neutralized with 3 M Tris-HCl, pH 8.8, and re-precipitated with 1 M CaCl_2_. This second CaCl_2_ precipitate, containing recombinant DSP-PP_240_ and products of processing, was dissolved in 0.5 ml of 0.1 M EDTA. The dissolved sample (20 µl) was fractionated on a non-denaturing polyacrylamide gel (7.5%). PP_240_ protein was stained with Stains-All and analyzed by MS and MS/MS analyses as described below.

7.5% non-denaturing polyacrylamide gel samples were stained with Stains-All and then PP_240_ band was excised, transferred to a 96-well plate, and destained. The gel samples were then subjected to reduction and alkylation and then washed, dehydrated, and digested with chymotrysin. The peptides were extracted from the gel plugs with 2% aceonitrile and 1% formic acid. The extracted peptides (30 µl) were transferred to another 96-well plate, where 5 µl of matrix (α-Cyano) was added to the sample well. The samples were then vaporized to dryness and redissolved in 5 µl of 60% aceonitrile and 0.1% trifuoroacetic acid. Peptide samples were then spotted on a MALDI-TOF/TOF target plate for MS and MS/MS analyses. MS/MS, or tandem mass spectrometry, is a mass spectrometric method in which a peptide is fragmented, and the masses of the resultant fragment ions are recorded in a spectrum. The analyses were performed using the ABI 4800 MALDI-TOF/TOF (Applied Biosystems, Foster City, CA) at the Michigan Protein Consortium. Searches for homologies between the amino acid sequences obtained and those of other known proteins in GenBank™, GenPept, and SwissProt were performed using BLAST software. The Michigan Proteome Consortium provided proteomics data at the University of Michigan.

### Preparation of 0-day to 3-day Conditioned Medium (CM_0–3d_)

Sf9 cells cultured at 28°C in T-25 flasks in 5 ml of Grace’s medium with 10% FBS and 50 µg/ml of Gentamycin were infected with DSP-PP_240_ virus for 3 days. A mock infection served as a control. Conditioned medium (CM) was harvested by centrifugation (500×g). CM containing DSP-PP_240_ precursor protein was designated “CM_0–3d_ with viral infection” and CM from uninfected Sf9 cells was designated “CM_0–3d_ without viral infection”.

### Preparation of 3-day to 7-day Conditioned Medium (CM_3–7d_)

Sf9 cells culture and infection with DSP-PP_240_ virus as described above. After three day viral infection, the conditioned medium was removed and discarded. Cells were then gently washed with 1×PBS and cultured in 5 ml of fresh Grace’s medium with10% FBS and 50 µg/ml Gentamycin for an additional 4 days (from day 3 to day 7 after infections) at 28°C. Then the CM, containing largely uncleaved DSP-PP_240_ precursor protein, was collected on day 7 by centrifugation (500×g) and designated CM_3–7d_.

### Testing for Proteolytic Activity in Condition Medium of Sf9 Cells

CM_3–7d_ (5 ml) from virus-infected cells was incubated with CM_0–3d_ (5 ml) from uninfected cells for different time periods at 28°C. The CM_3–7d_ from infected cells contained uncleaved DSP-PP_240_ while the CM_0–3d_ from uninfected cells contained endogenous proteolytic activity secreted by Sf9 cells. In parallel, CM_3–7d_ (5 ml) from virus infected cells was incubated with Grace’s medium containing 10% FBS and 50 µg/ml Gentamycin (5 ml) as a control. At each time point, 1 ml samples of mixture was processed using a standard purification protocol [15]. Briefly, 1 ml medium was adjusted to 5% TCA, in which DSP-PP_240_, DSP_430_ and PP_240_ are soluble but other proteins in conditioned medium are precipitated, incubated at room temperature for 2 min and then centrifuges in an Eppendorf rotor at 12,000 rpm for 5 min at room temperature. The supernatant was neutralized with one-fifth volume of 3 M Tris-HCl pH 8.8 and then precipitated by adding one-tenth volume of 1 M CaCl_2_ and centrifuging in an Eppendorf rotor at 12,000 rpm for 5 min at room temperature. The pellet from 1 ml medium was resuspended in 100 µl of 0.1 M EDTA. 15 µl of resuspended samples were resolved onto native PAGE as described previously [15], and the gels were fixed and stained with Stains-All to visualize DSP-PP_240_ and the cleavage products DSP_430_ and PP_240_. Stained gels were subjected to quantitative densitometry. Because the PP_240_ portion of DSP-PP_240_ is responsible for the majority of staining by the Stains-All reagent, percentage of protein processing was defined as PP_240_/(PP_240_+ DSP-PP_240_).

### Identification of Tolloid-related-1 Protein mRNA in Sf9 Cells

**Figure 1:**
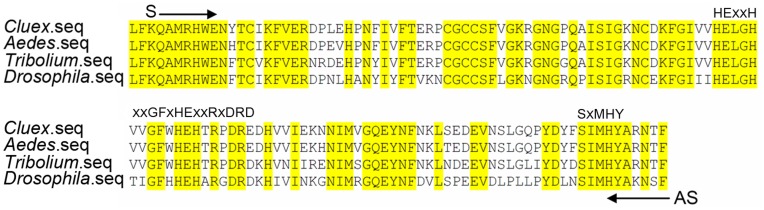
Alignment of consensus sequences of tolloid proteins from *Culex qunquefasciatus, Aedes aegypti, Tribolium castaneum and Drosophila melanogaster.* Sequences of tolloid protein catalytic domains from the above four species were aligned to identify consensus sequences to be used for designing primer sequences. Consensus peptide sequences are indicated by yellow highlights. Arrows represent the positions of peptide sequences QAMRHWE and IMHYA(R/K)N(T/S) that were used to make sense primer (S) and anti-sense primer (AS). These S and AS primers encompass sequences for the Zn-binding motif (HExxHxxGFxHExxRxDRD) and for another conserved region, the Met-turn (SxMHY).

We aligned tolloid protein sequences from *Drosophila melanogaster* (GeneBank accession number NM_079763), *Culex quinquefasciatus* (GeneBank accession number XM_001861486), *Aedes aegypti* (GeneBank accession number XM_001653501) and *Tribolium castaneum* (GeneBank accession number XM_965069), and obtained consensus sequences, [i.e., QAMRHWE and IMHYA(R/K)N(T/S)) as shown in Fig. 1. Sense and anti-sense oligonucleotide primers were generated according to these consensus peptide sequences. Total RNA from Sf9 cells infected with DSP-PP_240_ virus for 3 days was extracted with TRIzol® Reagent (Invitrogen, Carlsbad, CA). Using RT-PCR, tolloid-related-1 cDNA fragments were generated from this RNA preparation. After cloning into a TOPO cloning vector (Invitrogen, San Diego, CA), candidate clones were identified by PCR and confirmed by DNA sequencing. The cDNA sequences were then used to search the NCBI non-redundant protein sequence database for homologous peptide sequences.

### BMP1 Cleavage of DSP-PP_240_

CM_3–7d_ from virus infected cells (5 ml) was incubated at 28°C with an equal volume of unconditioned (fresh) Grace’s medium in the presence of 33 ng/ml or 170 ng/ml of recombinant human BMP1 or with no enzyme addition for various times from 1 h to 72 h as indicated in the corresponding figure. At each of the time points, 1 ml of mixed medium was processed as described above. 15 µl of each resuspended sample was analyzed by native PAGE, fixed and stained with Stains-All and the percentage of protein processed was determined as described above.

### Quantification of DSP-PP_240_ and PP_240_

Stained gels were dried, scanned to produce TIFF files and NIH ImageJ was used to quantify intensity of Stains-All stained bands. The stained protein band images were converted to grayscale and inverted and rectangular areas were used to integrate the intensity of the DSP-PP_240_ and PP_240_ bands. These intensity values were then used to calculate the ratio of PP_240_/(DSP-PP_240_+PP_240_). Each experiment was conducted at least 2 times and in most cases 3 or more times with comparable results. Plotted PP_240_/(DSP-PP_240_+PP_240_) ratios expressed as a percentage, represent the mean of at least two experiments and error bars represent the standard deviation of the mean. Data were analyzed and plotted using KaleidaGraph (Synergy Software, Reading, PA).

## Results

### DSP-PP_240_ Expressed in Sf9 Cells is Cleaved in Conditioned Medium at the Physiological Processing Site: SMQG^447^↓D^448^DPN

**Figure 2:**
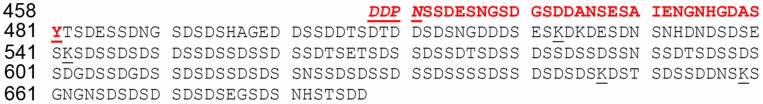
Mass spectrometric analyses of chymotrypsin digested mature PP_240_. The location of MS/MS identified peptide sequence for PP_240_ recombinant protein is labeled in red. The chymotrypsin cleavage site is located C-terminal to the underlined Tyr residue (Y).

Previously, we showed that full-length DSP-PP_240_ can be expressed in Sf9 cells using a baculovirus vector and that the precursor is partially processed into stable fragments of sizes expected for the physiological products DSP_430_ and PP_240_ [15]. Based on mass spectrometric analysis, we identified a 7.7 kDa polyacrylamide gel band from trypsinized recombinant PP_240_ that likely represented a 76 residue peptide containing the presumed N-terminal sequence of PP_240_. Due to its large molecular mass, it was not possible to determine the sequence of the peptide directly [15]. To overcome this problem, we used chymotrypsin digestion of baculovirus-expressed PP_240_, which would be expected to produce a 34 amino acid peptide corresponding to the N-terminus of PP_240_ (DSP-PP_240_ residues 448–481) if the precursor was cleaved after G^447^. MS/MS analysis of this chymotryptic peptide provided the entire expected sequence: DDPNSSDESNGSDGSDDANSESAIENGNHGDASY. This finding demonstrates that baculovirus-produced recombinant PP_240_ protein starts with DDPN, which corresponds to the N-terminal sequence of phosphophoryn purified from dental matrix (see Fig. 2). Thus cleavage of DSP-PP_240_ precursor protein in the baculovirus expression system occurs at the physiological site, generating stable fragments that do not undergo further cleavage.

### Processing of DSP-PP_240_ into DSP_430_ and PP_240_ Occurs after Secretion into Conditioned Medium and is Catalyzed by a Proteolytic Activity Secreted by Sf9 Cells

#### Continued cleavage of DSP-PP_240_ in CM_0–3d_ into DSP_430_ and PP_240_ during additional 3-day incubation at 28°C

**Figure 3:**
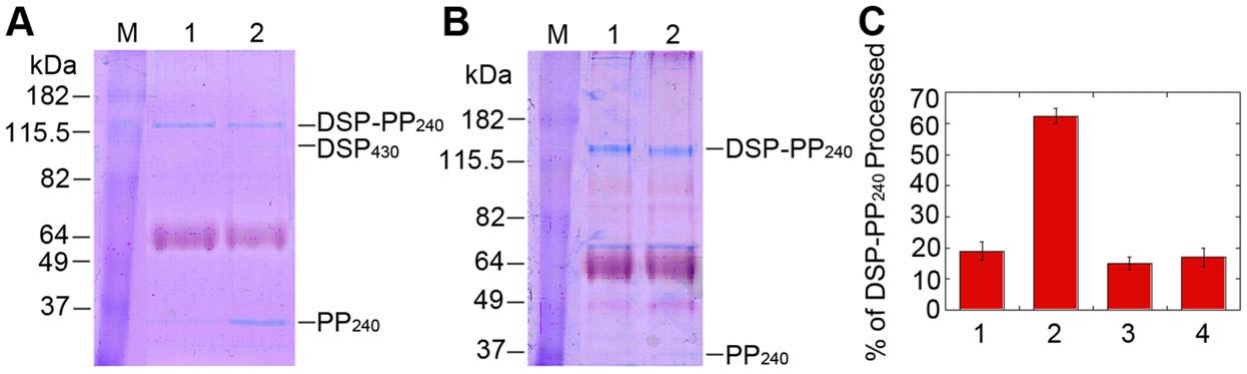
Processing of baculovirus-encoded DSP-PP_240_ in conditioned medium requires an activity that is only secreted by Sf9 cells early but not late after infection. (A) Processing of DSP-PP_240_ in conditioned medium of virus-infected Sf9 cells. Sf9 cells were infected with baculovirus containing the DSP-PP_240_ cDNA. In lane 1, 3 days after infection, CM_0–3d_ was collected and processed for native PAGE and Stains-All staining as described in Materials and Methods. In lane 2, CM_0–3d_ was incubated for an additional 3 days at 28°C before processing for PAGE. M represents protein size markers. (B) Medium conditioned by infected cells 3 to 7 days after infection lacks processing activity. 3 days after infection of Sf9 cells with baculovirus containing DSP-PP_240,_ the medium was replaced with fresh Grace’s medium containing 10% FBS and 50 µg/ml Gentamycin and the cells were cultured for an additional 4 days. In lane 1, the resulting conditioned medium, CM_3–7d_ was collected and processed for native PAGE and Stains-All staining. In lane 2, CM_3–7d_ was incubated for an additional 3 days at 28°C before processing. M represents protein size markers. (C) Quantification of DSP-PP_240_ precursor processing. Using NIH J image, we measured the image intensity of DSP-PP_240_ and PP_240_ bands in gels shown in panels A & B and two other similar gels. Calculation of percent processing was as described in Materials and Methods. Because DSP staining was very weak by Stains-All staining, total density was defined as the sum of the DSP-PP_240_ and PP_240_ image densities. Numbers 1–4, respectively, refer to panel A lane 1, panel A lane 2, panel B lane 1 and panel B lane 2. Error bars represent standard deviation of at least duplicate samples.

To determine the mechanism of cleavage of DSP-PP_240_ expressed in Sf9 cells, we first examined the time course of cleavage. In the conditioned medium from Sf9 cells infected with the DSP-PP_240_ virus for three days (CM_0–3d_), only a small fraction of DSP-PP_240_ was cleaved into mature PP_240_ (Fig. 3A, lane 1, Fig. 3C). After an additional 3 day incubation, DSP-PP_240_ precursor protein underwent further cleavage, resulting in accumulation of much higher levels of DSP_430_ and PP_240_ (Fig. 3A, lane 2). Because DSP_430_ is weakly stained by Stains-All relative to PP_240_, the DSP_430_ band was barely visible, and PP_240_ alone was therefore used as a quantitative marker for DSP-PP_240_ protein cleavage. Quantification of DSP-PP_240_ and PP_240_ bands showed that 18% of the DSP-PP_240_ was cleaved to PP_240_ in the CM_0–3d_ sample and that this increased to 60% after additional 3 day incubation, indicating that significant additional DSP-PP cleavage occurred during the extended 3 day incubation period. Thus DSP-PP_240_ cleavage largely occurred after secretion into conditioned medium and that the conditioned medium contained a proteolytic activity that was active during the additional 3 day incubation.

#### DSP-PP_240_ in CM_3–7d_ undergoes little cleavage into DSP_430_ and PP_240_ after an additional 3-day incubation at 28°C

To determine whether DSP-PP_240_ production continued beyond the third day after infection, the culture medium of Sf9 cells infected with the DSP-PP_240_ virus for 3 days was replaced with fresh medium and incubation was continued for 4 days. When this 3-to-7 day conditioned medium (CM_3–7d_) was analyzed, even larger amounts of DSP-PP_240_ were present than that in CM_0–3d_ (compare Fig. 3A, lane 1 to Fig. 3B, lane 1), indicating that DSP-PP_240_ production continued during the additional 4 day incubation period. However, the PP_240_ cleavage product band was barely visible, representing only about 15% cleavage of the precursor (Fig. 3C). Even after an additional 3 day incubation in the same medium, the PP_240_ band was still barely visible (Fig. 3B, lane 2), implying that no significant additional cleavage occurred (Fig. 3C). These results demonstrate that, whereas DSP-PP_240_ processing was dramatically increased with the extended 3 day incubation of the CM_0–3d_ sample, DSP-PP_240_ secreted into fresh medium after 3 days of infection (the CM_3–7d_ sample) was largely uncleaved and remained so even when incubated for an additional 3 days (Fig. 3B). Thus the CM_3–7d_ sample appeared to lack the DSP-PP processing activity.

#### CM_0–3d_ from uninfected Sf9 cells contains a proteolytic activity that promotes DSP-PP_240_ precursor protein cleavage

**Figure 4:**
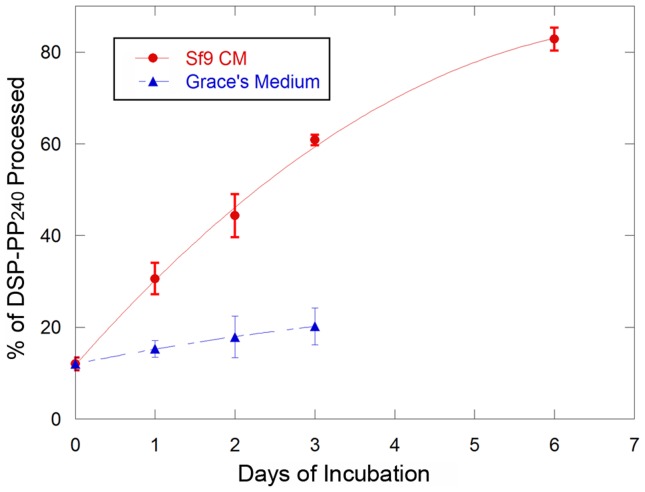
DSP-PP_240_ is cleaved by an activity secreted into conditioned medium by uninfected Sf9 cells. CM_3–7d_ from virus infected cells (containing most intact DSP-PP_240_), was mixed with an equal volume of 3d conditioned medium from uninfected Sf9 cells or with an equal volume of unconditioned Grace’s medium as described in Materials and Methods. At the times shown, medium samples were processed for native PAGE and Stains-All staining as described in Materials and Medthods. Percent processing of DSP-PP_240_ was determined as described in Materials and Methods. Error bars represent standard deviation of at least duplicate samples.

Because DSP-PP_240_ present in the CM_3–7d_ media did not undergo cleavage even during extended incubation periods, we used this DSP-PP_240_ sample as substrate to test for the production of a DSP-PP_240_ processing activity by conditioned medium of uninfected Sf9 cells. To do this, we mixed (1∶1) CM_3–7_ from infected cells with medium conditioned by incubation for three days with uninfected Sf9 cells and incubated the mixture at 28°C. CM_3–7d_ from infected cells was incubated with Grace’s medium as a control. Fig. 4 shows that, as a function of time, incubation of CM_3–7d_ from infected cells with CM_0–3d_ medium from uninfected Sf9 cells resulted in progressive cleavage of DSP-PP_240_ to PP_240_ that was roughly linear for the first 3 days of incubation and reached near completion (∼80% cleavage) after 6 days (Fig. 4, upper curve). Little cleavage was seen in the control containing unconditioned Grace’s medium (Fig. 4, lower curve). Therefore, Sf9 cells secrete an activity that correctly processes DSP-PP_240_, but secretion of this activity is greatly diminished or suppressed within 3 days after virus infection.

### Identification of a Tolloid-related-1 Transcript in Sf9 Cells

Mammalian BMP1 was reported to be a protease capable of cleaving several extracellular matrix proteins, including the pro-α1 precursors of type I, type II, type III, and type VII collagen, the pro-α2 precursors of type I and type V collagen, the human prolysyl oxidase, and DMP1 [11], [16]–[21]. Because the cleavage site of DMP1 exhibits high sequence similarity to the putative cleavage site in DSP-PP [11], we asked whether a BMP1 equivalent protein was present in Sf9 cells.

**Figure 5:**
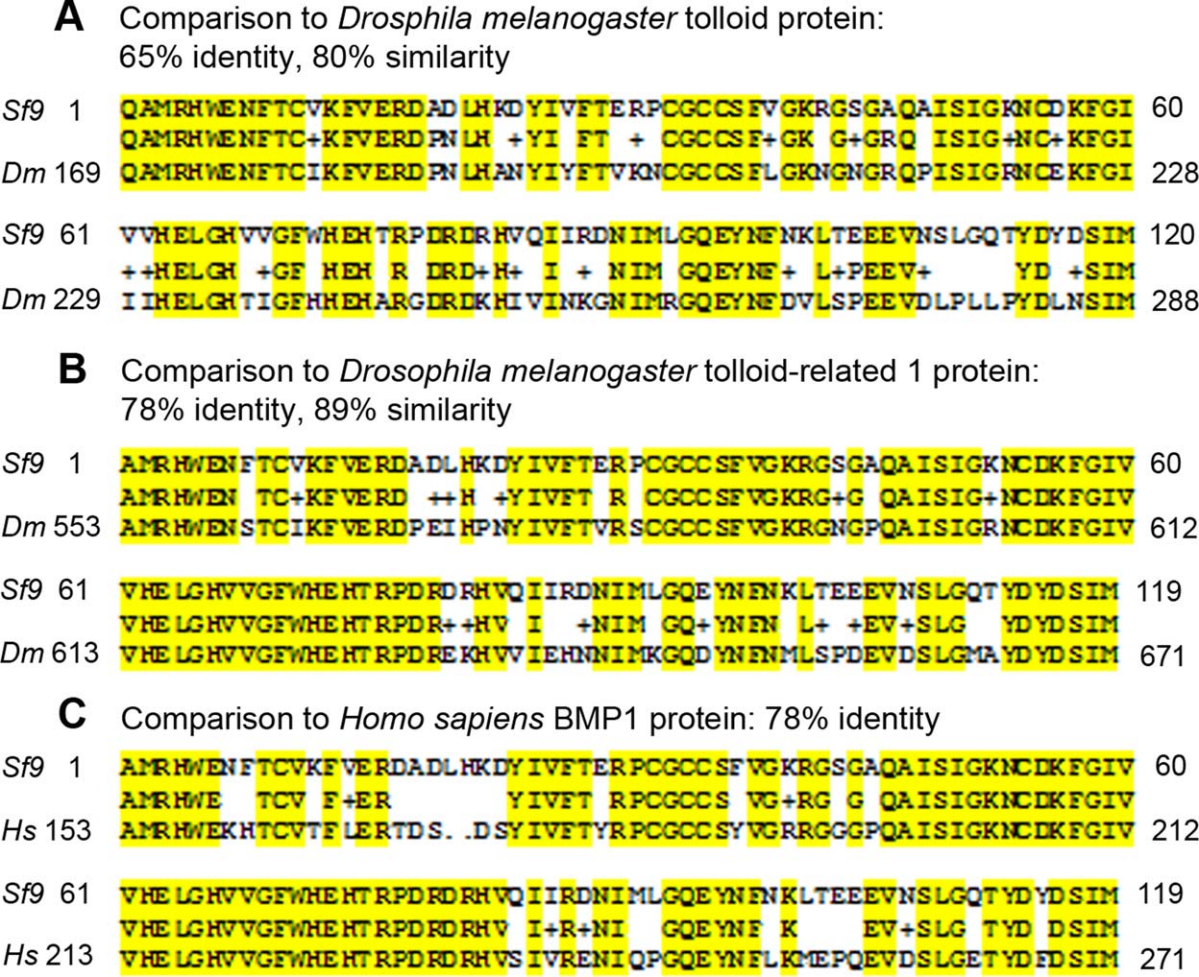
Partial Sf9 TLR1 sequence aligned with *Drosophila* TLD/TLR1 protein and *Homo sapiens* BMP1. The upper in each case represents the cloned Sf9 TLR1 sequence and lower line the test sequence. (A) The peptide sequence derived from the cloned Sf9 *tlr1* cDNA shared 65% sequence identity with *Drosophila melanogaster* TLD. (B) The peptide sequence derived from the cloned Sf9 *tlr1* cDNA shared 78% sequence identity with *Drosophila melanogaster* TLR1. (C) The peptide sequence derived from the cloned Sf9 *tlr1* cDNA shared 78% sequence identity with *Homo sapiens* BMP1.

The proteins in *Drosophila melanogaster* most closely related to human BMP1 are *Drosophila* tolloid (TLD) and tolloid-related-1 (TLR1). We therefore investigated whether Sf9 cells contain tolloid-related-1 mRNA sequences. We first obtained consensus sequences by aligning tolloid sequences from *Drosophila melanogaster, Culex quinquefasciatus, Aedes aegypti* and *Tribolium castan*. Using this alignment, we designed consensus primers corresponding to the most highly conserved regions of the catalytic domains of these enzymes and performed RT-PCR on Sf9 cell mRNA. We obtained a partial cDNA sequence encoding an open reading frame with 65% peptide sequence identity to *Drosophila* tolloid protein (TLD) and 78% peptide sequence identity to *Drosophila* tolloid-related-1 protein (TLR1). Because of greater similarity to TLR1, we propose that this clone represents a partial cDNA of a *Spodopteria frugiperda tlr1* mRNA. This cDNA encodes part of the astacin protease domain with the known consensus sequence HExxHxxGFxHExxRxDRD containing the zinc-binding motif, and another conserved region, SxMHY, the so-called Met-turn (see Fig. 5).

**Figure 6:**
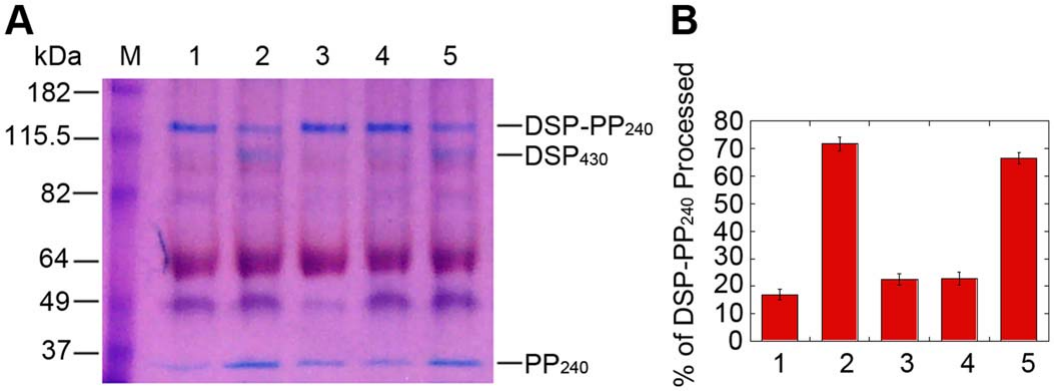
Processing of DSP-PP_240_ in conditioned medium is Zn-dependent. (A) Stains-All staining of DSP-PP_240_ cleavage. Lane 1: CM_0–3d_ of virus-infected cells without further incubation. Lane 2: CM_0–3d_ from virus-infected cells incubated for an additional 3days. Lane 3: Same reaction as lane 2 except with addition of EGTA (22 mM). Lane 4: Same reaction as lane 2 except with addition of 1mM 1,10-phenanthroline. Lane 5: Same reaction as lane 2 except with addition of 1 mM 1,10-phenanthroline and 1 mM ZnCl_2_. M represents size marker. (B) Quantification of DSP-PP_240_ processing shown in panel A and two similar gels. Numbers 1–5 correspond to lanes 1–5. Error bars represent standard deviation of the mean.

Because proteases in the astacin/TLD/BMP1 family are Zn-metallopeptidases, we tested EGTA and 1,10-phenanthroline [22], [23], both strong Zn^++^ chelators capable of inhibiting Astacin-type metallopeptidase activities, to determine whether these reagents could inhibit DSP-PP_240_ cleavage. As shown in Fig. 6, both 22 mM EGTA and 1 mM 1, 10-phenanthroline inhibited DSP-PP_240_ cleavage to DSP_430_ and PP_240_ in Sf9 cell-free medium. Moreover, inclusion of 1 mM ZnCl_2_ prevented inhibition by 1 mM 1,10-phenanthroline (Fig. 6A&B, compare lanes 4 and 5), demonstrating that DSP-PP_240_ processing activity secreted by Sf9 cells is a Zn-requiring enzyme.

**Figure 7:**
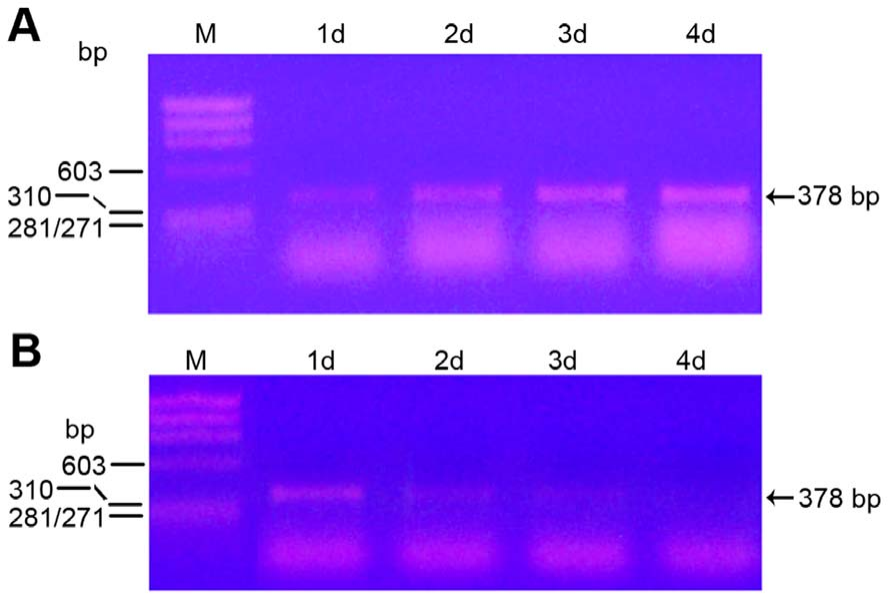
*Tlr1* mRNA expression. At the indicated time point, medium was removed, Sf9 cells were washed with PBS, and total RNA was extracted with RNAzole. Then reverse transcription was performed to generate a cDNA pool. *Tlr1* sense and antisense primers were used to detect the presence of *tlr1* mRNA at various time points. (A) cDNA pools (100 µl) were generated from 2 µg total RNA from uninfected Sf9 cells and 1 µl was used for PCR analyses. Lane 1: ϕx174 Hae III size marker. Lane2: 1d Sf9 cells. Lane3: 2d Sf9 cells. Lane 4: 3d Sf9 cells. Lane 5: 4d Sf9 cells. (B) cDNA pools (100 µl) were generated from 2 µg total RNA from Sf9 cells infected with baculovirus containing DSP-PP_240_ cDNA and 2 µl was used for PCR analyses. Lane 1: ϕ×174 Hae III size marker. Lane 2: 1d viral infection. Lane 3: 2d viral infection. Lane 4: 3d viral infection. Lane 5: 4d viral infection. The arrow indicates the position of the 378 bp partial *tlr1* PCR fragment.

We next tested whether *tlr1* mRNA was expressed by uninfected and DSP-PP_240_ viral infected Sf9 cells. Using the sense and antisense primers specific for Sf9 *tlr1* cDNA, we performed RT-PCR on RNA from uninfected and DSP-PP_240_ viral infected Sf9 cells. In Sf9 cells without viral infection, *tlr1* mRNA expression increased steadily from day 1 to day 4 (Fig. 7A). In Sf9 cells infected with DSP-PP_240_ virus, *tlr1* mRNA expression was highest at 1 day post-infection but dropped dramatically from day 2 to day 4 post-infection (Fig. 7B). The decrease seen in expression of Sf9 *tlr1* mRNA after virus infection is consistent with the lack of secretion of DSP-PP_240_ processing activity by 3 days after viral infection (Fig. 3B).

### Cleavage of DSP-PP_240_ Precursor Protein by BMP1

**Figure 8:**
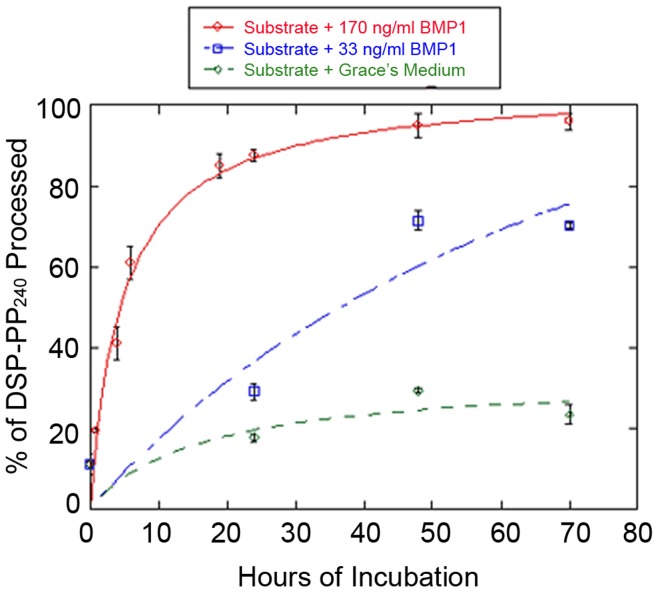
DSP-PP_240_ is accurately cleaved by human BMP1. CM_3–7d_ from virus infected cells containing mostly unprocessed DSP-PP_240_ precursor protein was incubated with an equal volume of Grace’s medium alone or Grace’s medium containing 33 ng/ml or 170 ng/ml of human BMP1 for the indicated times at 28°C. At each time point, 1 ml samples were processed for native PAGE and Stains-All staining and gels quantified as describe in Materials and Methods. The data were fit to the Michaelis-Menton equation using KaleidaGraph. Error bars represent standard deviation of at least duplicate samples.

We next tested whether purified BMP1 could cleave DSP-PP_240_. For these studies we utilized CM_3–7d_ medium samples, which contained significant quantities of stable DSP-PP_240_ precursor protein as substrate. Fig. 8 shows the kinetics of DSP-PP_240_ cleavage by BMP1 added to the CM_3–7d_ substrate at concentrations of 33 ng/ml or 170 ng/ml. BMP1 cleaved DSP-PP_240_ precursor protein in a concentration-dependent manner yielding mature PP_240_, while control incubation with Grace’s medium yielded minimal cleavage.

Taken together, the results presented here show clearly that DSP-PP_240_ precursor secreted by viral infected Sf9 cells is cleaved after secretion, at the known physiological cleavage site, by an endogenous Zn-metalloproteinase activity secreted by Sf9 cells to yield DSP_430_ and PP_240_ proteins. We have shown that BMP1 is also capable of correct cleavage of DSP-PP_240_ produced in the baculovirus system. We have shown further that Sf9 cells express an mRNA encoding the BMP1 homolog, TLR1. A reasonable interpretation of these results is that the specific cleavage of DSP-PP_240_ in the Sf9 cells expression system represents the action of a highly conserved processing enzyme, TLR1, on the mammalian substrate.

## Discussion

Expression of the DSP-PP precursor protein is essential for normal tooth development due to requirement for PP in dental matrix mineralization. As a result, elucidating the mechanism and regulation of DSP-PP maturation is fundamental to understanding matrix formation. As shown here, baculovirus expression of DSP-PP_240_ can provide a valuable model with which to address key questions about the maturation of DSP-PP. The baculovirus expression system permits the purification of substantial quantities of DSP-PP_240_ precursor for biochemical analysis of processing. Like the precursor produced by odontoblasts, DSP-PP_240_ produced in Sf9 cells is extensively glycosylated and phosphorylated (Ritchie HH, unpublished results).

Previously [15], we found that recombinant DSP-PP_240_, isolated using TCA precipitation, neutralization, calcium precipitation, and EDTA resuspension followed by preparative native PAGE, underwent spontaneous cleavage into DSP_430_ and PP_240_ when incubated at 37°C. We considered it unlikely that a contaminating protease in gel-purified DSP-PP_240_ was responsible for cleavage because of the harsh, denaturing conditions used in the purification. Indeed, the low pH and chelation steps should have inactivated Zn-dependent peptidases. Regardless of these results, we have shown here that DSP-PP_240_ is secreted into the conditioned medium intact, where it undergoes accurate and specific processing at the physiological cleavage site by a Zn-metalloproteinase secreted by Sf9 cells to generate stable fragments that correspond to the physiological cleavage products DSP_430_ and PP_240_.

In this study, we compared the fate of DSP-PP_240_ secreted into conditioned medium in the first 3 days after viral infection (CM_0–3d_) to that of DSP-PP_240_ secreted into freshly added medium during days 3–7 after infection (CM_3–7d_). Surprisingly, while significant amounts of mature DSP_430_ and PP_240_ were generated by incubation of the CM_0–3d_ sample, little, if any cleavage of DSP-PP_240_ occurred in the CM_3–7d_ sample on extended incubation, suggesting that a processing enzyme secreted by Sf9 cells within the first 3 days after infection, was no longer secreted after the first three days. Using CM_3–7d_ media, which contained stable DSP-PP_240_, we could test whether a processing enzyme was secreted by uninfected Sf9 cells. We were able to demonstrate that CM_0–3d_ media from uninfected Sf9 cells contained a Zn-dependent proteolytic activity that cleaved the DSP-PP_240_ into DSP_430_ and PP_240_ (Fig. 4 and Fig. 6).

Sf9 cells are routinely used for expression of recombinant proteins encoded by baculovirus vectors. Our identification of a partial *tlr1* cDNA from Sf9 cells is a new finding that may prove useful to others interested in examining the cleavage of Sf9-produced recombinant proteins. Although we do not have definitive evidence that the DSP-PP_240_ processing activity is Sf9 TLR1, the fact that the activity is secreted and Zn-dependent and that it cleaves DSP-PP_240_ selectively at the physiological cleavage site makes it likely that this is the case. Moreover, the decline in expression of *tlr1* mRNA after infection (Fig. 7B), which may be due to baculovirus inhibition of the endogenous gene expression, paralleled the decline seen in DSP-PP_240_ cleavage activity in the conditioned medium (Fig. 3B).

As we have shown here, the decrease in endogenous processing activity after infection can be used to produce unprocessed DSP-PP_240_ that can be used as substrate in studies using exogenously added protease. Using this approach, we have shown that BMP1 can accurately process baculovirus-expressed DSP-PP_240_ to produce stable PP_240_ and DSP_430_. These data are consistent with BMP1, or one of the related enzymes TLL1 or TLL2, being the physiological processing enzyme for DSP-PP. BMP1 belongs to the astacin family of metalloproteinases [24], [25] and is present in many tissues [26]. BMP1 was reported to be involved in the cleavage of several extracellular matrix proteins such as the pro-α1 precursors of type I, type II, type III, and type VII collagen, the pro-α2 precursors of type I and type V collagen [16]–[20], and the human prolysyl oxidase [21]. In bone and teeth, as well as in non-mineralized tissues, BMP1 is required for cleavage of such extracellular matrix proteins as procollagen, to provide mature proteins that can then go on to effect both hard and soft tissue maturation. DSP-PP is also found in non-mineralized tissues (i.e., kidney, hair follicle, salivary gland and lung) that also contain BMP1, suggesting a role for BMP1 cleavage in precursor maturation in these tissues as well.

Taken together, our results suggest that the specific and physiologically accurate cleavage of DSP-PP_240_ in the Sf9 cell system represents the action of a highly conserved processing enzyme.

Based on our results with the Sf9 cell system, we favor a model in which DSP-PP is cleaved after secretion into dentin matrix by BMP1 (or TLL1 or TLL2). This is suggested by the observation that although DSP-PP_240_ and the Zn-dependent processing activity are co-expressed and co-secreted by Sf9 cells, the majority of DSP-PP_240_ processing cannot be occurring until after secretion. Key questions raised by this model that can be addressed in the baculovirus system are why processing is delayed until secretion and how cleavage is prevented until after secretion if DSP-PP and BMP1 are co-secreted. Delay of processing may be necessary if the DSP portion of the precursor is required to prevent premature oligomerization of PP, which has been found to form large paracrystalline aggregates when expressed without the DSP portion [27]. Intracellular processing may be prevented if DSP-PP_240_ undergoes a conformational change upon secretion, possibly due to high extracellular calcium that promotes efficient cleavage by BMP1 or a related enzyme. Finally, it is of interest to know whether interaction with extracellular calcium protects mature DSP and PP from further proteolysis.
